# Effects of Selenium *Auricularia cornea* Culture Supplementation on Growth Performance, Antioxidant Status, Tissue Selenium Concentration and Meat Quality in Growing-Finishing Pigs

**DOI:** 10.3390/ani11092701

**Published:** 2021-09-15

**Authors:** Ying Ju, Mingzhi Liu, Liling Huang, Yanglan Luo, Liangliang Qi, Jianqiang Ye, Xiaojian Wu, Naixin Cao, Jianing Bo, Xuzhou Liu, Yong Yan, Yu Li

**Affiliations:** 1 Institute of Microbiology, Guangxi Academy of Agricultural Sciences, Nanning 530007, China; 13578935348@163.com (Y.J.); gaashll@126.com (L.H.); gaaslyl@126.com (Y.L.); gaasqll@126.com (L.Q.); gaasyjq@126.com (J.Y.); gaaswxj@126.com (X.W.); gaascnx@126.com (N.C.); 2 Guangxi Crop Genetic Improvement and Biotechnology Laboratory, Guangxi Academy of Agricultural Sciences, Nanning 530007, China; 3 Lishu Blackland Healthy Food Co., Ltd., Siping 136599, China; jlgreenlzy@126.com (M.L.); jlgreenbjn@126.com (J.B.); 4 Engineering Research Center of Chinese Ministry of Education for Edible and Medicinal Fungi, Institute of Mycology, Jilin Agricultural University, Changchun 130118, China; infungi@163.com

**Keywords:** Selenium *Auricularia cornea* culture, growth performance, antioxidant status, growing-finishing pigs, meat quality

## Abstract

**Simple Summary:**

Selenium *Auricularia cornea* culture (SAC) is a dried product via full fermentation, containing organic-Se, *Auricularia cornea* (AC) mycelium, and various metabolites of AC. The objective of this study was to evaluate whether SAC could effectively improve the health, growth, meat quality, and oxidative stability of meat in growing-finishing pigs. Currently, dietary SAC supplementation positively impacts growth performance and oxidative stability of fresh meat.

**Abstract:**

Selenium *Auricularia cornea* culture (SAC) is a new source of organic selenium. Two experiments were conducted to determine the available energy of SAC fed to pigs and to evaluate the effects of dietary SAC supplementation on growth performance, serum biochemical profiles, fecal short chain fatty acids (SCFA), meat quality, tissue selenium concentration, and oxidative stability of fresh meat in growing-finishing pigs. In Experiment (Exp.) 1, 12 barrows with average body weight (BW) of 42.40 ± 5.30 kg were randomly allotted to two groups and fed the basal diet and SAC-supplemented diet, individually. In Exp. 2, 96 growing-finishing pigs (BW: 91.96 ± 7.55 kg) were grouped into four dietary treatments; each treatment contained six replicates with four pigs per replicate. The four treatments fed a control diet and three experimental diets supplemented with 0.6%, 1.2%, and 2.4% SAC, respectively. The trial lasted for 45 days. The results revealed that digestible energy (DE) of SAC was 11.21 MJ/kg. The average daily gain (ADG) was improved in pigs fed 1.2% and 2.4% SAC during day 24 to 45 and the overall period. Dietary 1.2% and 2.4% SAC supplementation had a lower F/G (*p* < 0.05) than the control diet during different stages. Dietary SAC supplementation increased fecal butyrate contents (*p* < 0.05), and pigs fed 1.2% and 2.4% SAC diets had a higher MCT1 mRNA expression (*p* = 0.04) in the colon. Pigs fed 2.4% SAC had higher GSH-Px contents (*p* < 0.05) in serum, liver, and *longissimus dorsi* muscle (LDM) than those in the control group. The 2.4% SAC-supplemented group revealed a higher Se content (*p* < 0.05) in LDM and a lower MDA concentration (*p* < 0.05) in fresh meat during the simulated retail display on day six. In conclusion, this study suggested that SAC was more effective in improving growth, enhancing the antioxidant status, depositing Se in muscle, and increasing meat oxidative stability of pigs.

## 1. Introduction

*Auricularia* mushrooms, a member of the basidiomycetes, also known as wood ears, are a group of edible fungi that form a gelatinous fruiting body [1]. It is well documented that *Auricularia* are widely distributed in temperate, subtropical, and tropical regions, particularly in many northern temperate-zone countries, including China, Japan, Korea, and Thailand [2,3]. As one of the traditional Chinese edible and medicinal fungi, *Auricularia* has been confirmed to possess various biological activities [4].

It has been generally considered that *Auricularia* polysaccharides (AP) are the major bioactive substance. Various bioactivities of AP have been reported, including immunomodulatory, antioxidant, antitumor activities, hepatoprotective [5]. Wu et al. (2010) [6] found that AP could modulate the immune functions of aged mice by increasing the weights of the thymus and spleen. In the opinion of Miao et al. (2020) [5], AP could activate the innate immune system and effector cells, including T-lymphocytes, B-lymphocytes, macrophages, and natural killer cells to express cytokines, such as IL-1β, TNF-α, IL-6, and IL-10. Five water-soluble APs from different varieties were all observed to have scavenging activities against superoxide, 2,2-diphenyl-1-picrylhydrazyl (DPPH), and hydroxyl radicals [7]. A soluble AP was observed to down-regulate the serum levels of blood lipids in dietary-induced hypercholesterolemic rats to normal [8]. According to Miao et al. (2020) [5], the AP could inhibit the absorption of exogenous lipids by binding with cholate or lipid molecules in gastrointestinal tracts and promote total cholesterol (TC) metabolism. The hypolipidemic activities were determined by molecular weight, monosaccharide compositions, uronic acid concentrations, and chain conformations of AP. Wang et al. (2018) [9] measured the hepatoprotective effects of AP in the acute alcohol-induced alcoholic liver diseases (ALD), and AP showed increasing antioxidant activities, reducing lipid peroxidation, inhibiting the expression levels of inflammatory mediators, and preventing the alcohol-induced histopathological alterations. The biological activities may be attributed to the abundant glucose and xylose concentrations in AP.

Selenium is an essential nonmetallic micronutrient for all animals, plays vital roles in several physiological processes, and has potent anti-inflammatory, antioxidant, and antiviral effects [10]. The bioavailability, as well as the toxicological effect of Se, was associated with chemical forms. Organic Se possessed a potent antioxidant capacity that provided greater protection against oxidative damage and less toxicity than inorganic Se [11]. Several studies indicated that dietary Se supplementation could improve the meat quality of chicken [12,13]. Chen et al. (2019) [14] showed that Se supplementation could enhance the antioxidant status and meat quality of growing-finishing pigs.

To produce Selenium *Auricularia cornea* culture (SAC), a specific culture media containing sodium selenite was inoculated with the *Auricularia cornea* (AC) strain and processed via solid fermentation under humidity–temperature controlled conditions for 15 days. Subsequently, the fermented culture media was dried at 60 °C. AS AC could utilize the carbohydrates and proteins present in media via enzymolysis, they result in a variety of metabolic products, such as organic selenium, peptides, amino acids, oligosaccharides, and polysaccharides. The analyzed nutrient content of SAC is shown in Table 1.

Because of its abundant nutraceutical compounds, we hypothesized that SAC might be an effective feed additive that could improve the growth and antioxidant status of pigs. There was also a strong correlation between oxidative stress and meat quality [15]. Therefore, the purpose of the current study was to determine the digestible energy (DE), metabolizable energy (ME) contents of SAC and to evaluate the effects of SAC supplementation on growth performance, short chain fatty acid (SCFA) contents in feces, serum biochemical parameters, carcass characteristics, meat quality, and oxidative stability of fresh meat in growing-finishing pigs.

## 2. Materials and Methods

### 2.1. Animals, Experimental Design and Sample Collection

Experiment (Exp.) 1. Determination of digestible energy (DE) and metabolizable energy (ME) contents

A total of 12 crossbred barrows (Duroc × Landrace × Yorkshire; initial body weight: 42.40 ± 5.30) were selected and assigned to two dietary treatments in a completely randomized design. Each treatment had 6 replicates and 1 pig per replicate. Due to SAC’s low palatability, it could not be fed for a long period. Therefore, the energy content in SAC was determined according to the difference method described by Kong et al. (2014) [16]. Briefly, the experimental diet was formulated to replace a portion of the basal diet by SAC. Therefore, the treatment diets included a corn-based diet (basal diet) and an experimental diet formulated by replacing 25% of corn with SAC in the basal diet (Table 2). Each pig was individually placed in a metabolism crate equipped with a feeder, a nipple, and fecal collection trays. The room temperature was controlled between 24 and 28 °C. All pigs were provided ad libitum access to water. The feed was divided into two equal meals supplied at 9:00 AM and 5:00 PM. The daily feed was equivalent to 4% of their initial body weight (IBW) [16]. Trial pigs were allowed a 7 day period to adapt to metabolic crates, then fed experimental diets for 12 days. Feces and urine samples were collected individually during the last 5 days. The feed refusals and spillage were gathered, dried, weighed, and recorded daily. The fecal samples were gathered separately in each collection tray, then stored at −20 °C. The urine samples were collected in the buckets containing 50 mL 6 mol/L Hydrochloric acid (HCL) under the metabolic crate. About 10% of urine was stored at −20 °C daily [17]. In the end, all samples were thawed, pooled, homogenized, and subsampled. The fecal subsamples were dried in a drying oven at 65 °C for 72 h. Urine samples (4 mL) after dropping into crucibles with filter paper were then dried at 65 °C for 8 h. The SAC, diets, feces samples were ground to pass through a screen (1 mm) and mixed thoroughly before determination. [18].

Exp. 2. Growth trail of SAC fed to growing-finishing pigs

Ninety-six growing-finishing pigs (Duroc × Landrace × Yorkshire; IBW: 91.96 ± 7.55 kg) were stochastically divided into 4 dietary treatments with 6 replicated pens per treatment, including a control diet and 3 experimental diets supplemented with 0.6%, 1.2%, and 2.4% SAC, respectively. The up-limit concentration of dietary selenium in swine feed is 0.5 mg/kg according to regulations in the European Union (EU) and the Food and Drug Administration (FDA) in the USA [14]. In the current study, the calculated Se content in the 2.4% SAC diet was 0.13 mg/kg. The experimental diets were formulated based on the DE content estimated from Exp. 1. As shown in Table 3, all diets were formulated to meet the nutrient requirements of NRC (2012) [19]. The trial lasted for 45 days, during which the treatment diets were offered at 8:00 a.m. and 3:00 p.m. each day. The ambient temperature was controlled at 25–28 °C. Feed and water were provided ad libitum. The trial included two phases: growth phase (phase 1: day 0–23) and finishing phase (phase 2: day 24–45). The amount of feed offered and body weight (BW) was weighed and recorded on day 0, 23, and 45 to determine average daily gain (ADG), average daily feed intake (ADFI), and feed conversion ratio (F/G). Twenty-four fresh feces were collected by rectal palpation from pigs (one pig per pen) for short chain fatty acid (SCFA) analysis [20]. On day 45, the pigs were selected by the BW close to the average BW in each pen, and then the blood samples were obtained using precaval vein puncture into the vacuette tubes (10 mL). The serum sample was subsequently acquired at 4 °C, 3000 r/s for 15 min and stored at −20 °C.

At the end of the trial, a total of 24 pigs (one pig per pen) close to the average BW of each pen were picked up for further slaughter experiments. After fasting (12 h), all selected animals were killed humanely. The colonic tissue samples were obtained and then stored at −80 °C for mRNA expression analysis. The *longissimus dorsi* muscle (LDM) (200 g) between the 10th and 12th ribs on the right half of the carcass, liver, and kidney tissues were sampled and immediately stored at −20 °C for meat quality assessment.

### 2.2. Chemical Analysis and Calculation

The samples were assessed for ether extract (EE), dry matter (DM), crude protein (CP), ash, total phosphors, and calcium [21]. The contents of neutral detergent fiber (NDF) and acid detergent fiber (ADF) were determined by fiber analyzer equipment (2010, FOSS, Hillerød, Denmark), according to the procedure described by Van Soest et al. [22]. The gross energy (GE) concentrations in diets, feces, and urine were measured using an Automatic Isoperibol Oxygen Bomb Calorimeter (C2000, IKA, Königswinter, Germany). The quantification of Se was carried out using an inductively coupled plasma source mass spectrometry (ICP-MS, iCAP Q, Thermo, Waltham, MA, USA). The concentration of short chain fatty acid (SCFA) was analyzed according to Wu et al. (2017) [23] using an ion chromatography (883, Metrohm, Herisau, Switzerland). The antioxidant indices, including glutathione peroxidase (GSH-Px), superoxide dismutase (SOD), malondialdehyde (MDA), and total antioxidant capacity (T-AOC), were measured by assay kits (Jiancheng Ins., Nanjing, China). The levels of high density lipoprotein (HDL), low density lipoprotein (LDL), glucose (GLU), total cholesterol (TC), and triglyceride (TG) in serum were measured using an automatic biochemical analyzer (3100, Hitachi, Tokyo, Japan) by relevant kits (Jiancheng Ins., Nanjing, China).

The DE and ME contents of SAC was calculated by the following the equation [24]: DE_SAC_ (MJ/kg) = [DE_experimental diet_ − (100% − X%) × DE_basal diet_]/X%; ME_SAC_ (MJ/kg) = [ME_experimental diet_ − (100% − X%) × ME_basal diet_]/X%, where the DE_experimental diet_, DE_basal diet_, ME_experimental diet_, and ME_basal diet_ were digestible energy in experimental diet and basal diet and metabolizable energy in experimental diet and basal diet, respectively. The X% represented the proportion of SAC replacing energy material in the basal diet. The DE_experimental diet_, DE_basal diet_, ME_experimental diet_, and ME_basal diet_ were calculated as follows: DE (MJ/kg) = (GE_intake_ − GE_fece_)/DMI; ME (MJ/kg) = (GE_intake_ − GE_fece_ − GE_urine_)/DMI, where the GE_intake_, GE_fece_, GE_urine_, and DMI were gross energy intake, gross energy output in feces, gross energy output in urine, and matter intake, respectively.

### 2.3. Determination of Carcass Traits and Meat Quality

The carcass length, carcass weight, and marbling were measured. The loin eye area and backfat thickness were measured and calculated. Briefly, pigs’ carcasses were weighed and recorded individually to calculate the hot carcass weight after slaughter. Then, all carcasses were placed in a 4 °C chilling room. The carcass length was recorded as the distance between the first rib and the pubic bone [25]. The dressing percentage was calculated as dressing percentage (%) = 100 × carcass weight/live body weight. The loin eye area and backfat thickness were measured at the 10th rib according to Chinese Guidelines on Performance Measurement Technology and Regulations for pigs (2014) [26]. The loin eye area was determined as loin eye area (cm^2^) = loin eye height (cm) × loin eye width (cm) × 0.7. The thickness of the first rib, last rib, and last lumbar vertebra was recorded using a vemier caliper, and the backfat thickness was then calculated. The marbling scores were estimated by the National Pork Producers Council (NPPC) guidelines of the United States [27].

The meat quality, including meat color, pH value, shear force, cooling loss, and drip loss, was subsequently analyzed. Briefly, the values of L* (lightness), a* (redness), and b* (yellowness) were measured at 45 min postmortem and 24 h after storing in a 4 °C chilling room for 24 h by a tristimulus colorimeter (NR, Mingao, Nanjing, China). The pH values were also determined at 45 min and 24 h postmortem by a glass penetration pH electrode (IS400-SP, Mingao, Nanjing, China). To determine the shear force, the meat samples were previously cooked in a hot water bath (70 °C for 20 min). Then, the shear force was evaluated by a muscle tenderness meter (C-LM3, Bulader, Beijing, China) [28]. The steak sample was weighed in their raw state and then weighed after they had reached 80 °C in an oven, and the cooking loss was calculated [29]. The slice samples were hung in a bag at 4 °C for 24 h, individually, then the drip loss was calculated as the following equation: drip loss (%) = the amount of drip/initial meat weight.

### 2.4. Relative Quantification of MCT1 mRNA Expression

The monocarboxylate transporter 1 (MCT1) mRNA expression analysis was carried out by standard procedures [30,31]. The liquid nitrogen-frozen colonic samples were pulverized by a mortar and pestle. The total RNA was extracted by the Invitrogen TRIzol reagent (Thermo Fisher, Waltham, MA, USA), then the quality and quantity of RNA were determined on a spectrophotometer (ND-1000, Thermo Fisher, Waltham, MA, USA). The total RNA samples were reverse-transcribed into complementary DNA (cDNA), individually, by the Superscript II transcriptase (Invitrogen, Thermo Fisher, Waltham, MA, USA). The primers for MCT1 and β-actin were designed according to published sequences [31] and was shown in Table 4. The RT-PCR was conducted in a system (10 μL) containing 0.2 μL ROX Reference Dye, 1 μL cDNA template, 0.2 μL each of forward and reverse primers, and 5 μL SYBR Green mix. Subsequently, the qPCR was performed with general cycling conditions: pre-denaturation for 10 s (95 °C 40 cycles of amplification for 5 s (95 °Cand 20 s (60 °C) melting curve construction with a heating rate of 0.1 °C per second (from 60 °C to 99 °C) and fluorescence measurements.

### 2.5. Oxidative Stability of Fresh Meat during Simulated Retail Display

A total of 24 LMD samples (one loin per pen) were selected. Then, the 2.5 cm-thick, boneless, closely-trimmed meat chops were placed on Styrofoam trays individually and overwrapped with oxygen-permeable polyvinyl chloride (PVC) film. Subsequently, all packaged chops were stored in an open-topped display case maintained at a temperature of 4 °C and continuous fluorescent lighting conditions (TLD-T8 36 W, 6200 K). On day 0, 3, and 6, approximately 10 g of chops were obtained for MDA content analysis [32].

### 2.6. Statistical Analysis

The data of growth performance, serum biochemical profiles, SCFA, meat quality, tissue selenium concentration, and MDA contents in fresh meat were checked for outliers and normality by the PROC UNIVERIATE of version 9.2 SAS (SAS Inst. Inc., San Diego, CA, USA). The treatment group was the only fixed effect. The pig was treated as the experimental unit (pen was treated as the experimental unit when analyzing growth performance data). The outliers were recognized by cook’s distance and abandoned in data analysis. Subsequently, the data were analyzed by the PROC GLM procedure. GLM procedure computes least square means and least square mean differences for classification effects. A probability value of 0.05 was considered statistically significant.

## 3. Results

The DE and metabolizable energy (ME) values of SAC were 11.21 and 10.92 MJ/kg (as-fed basis), respectively (Table 5).

As shown in Table 6, pigs fed a 2.4% SAC diet had a higher final body weight (FBW) (*p* < 0.05) than the other three diets. Compared with the control diet, pigs fed diets supplemented with SAC showed a decreased F/G (*p* < 0.05) value during day 1 to 23. From day 24 to 45, 1.2% and 2.4% SAC supplemented in diets enhanced ADG and reduced F/G, compared with the control and 0.6% SAC diets (*p* < 0.01). During the overall period, pigs in 1.2% and 2.4% SAC treatments had the higher ADG (*p* < 0.05), and 0.6%, 1.2%, and 2.4% SAC treatments had the lower F/G than that in the control treatment (*p* < 0.05).

Compared with the control diet, dietary SAC supplementation significantly increased butyrate concentration (*p* < 0.05) on day 45 (Table 7).

**Figure 1:**
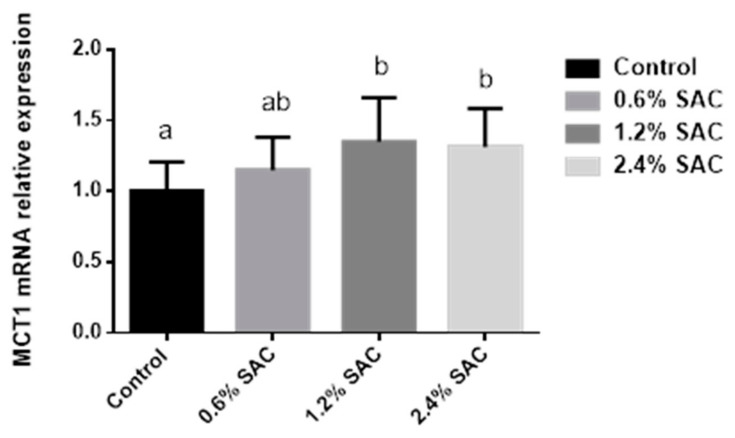
Relative MCT1 mRNA expression in the colon of pigs fed a control diet or diets supplemented with 0.6%, 1.2%, and 2.4% Selenium *Auricularia cornea* culture (SAC). Values are LS means (*n* = 6) with standard errors represented by vertical bars. ^a,b^ Least squares means within different superscripts differ (*p* < 0.05).

As shown in Figure 1, pigs fed 1.2% and 2.4% SAC diets showed a higher MCT1 mRNA expression (*p* = 0.04) in the colon than in the control diet.

As illustrated in Table 8, the GSH-Px contents (*p* < 0.05) in 1.2% and 2.4% SAC groups were remarkably higher than that in the control group on day 45. However, no differences in T-AOC, SOD, MDA, and the other serum indices were found in finishing pigs fed different levels of SAC.

As revealed in Table 9, dietary supplementation of 2.4% SAC significantly improved GSH-Px content (*p* < 0.05), and SAC supplemented at 1.2% and 2.4% markedly reduced MDA contents (*p* < 0.05) in the liver. The GSH-Px activity (*p* < 0.05) in 2.4% treatment was significantly higher in LDM than in the control treatment.

Finishing pigs fed 2.4% SAC showed a higher Se content (*p* < 0.05) in LDM compared with the other three treatments (Table 10). There was an increasing trend in liver Se content for pigs fed SAC diets (*p* = 0.09).

As shown in Table 11, no effects on carcass traits and meat quality were observed in pigs fed different levels of SAC.

As displayed in Table 12, compared with the control diet, pigs fed a 2.4% SAC diet had lower MDA content (*p* < 0.05) in fresh meat during the simulated retail display on day 6.

## 4. Discussion

The ME value is defined as the DE minus GE in urine and fermentation gases. The ME represents approximately 92–98% of the proportion of DE. Gas energy varies and is typically small for conventional diets fed to pigs (about 0.5% DE for growing-finishing pigs). It could be as high as 3% of DE in sows fed high-fiber diets. The GE value in urine is the primary factor defining the proportion of DE converted to ME [19]. According to the procedure by Adeola [23], we performed this study without evaluating gas losses on ME measurement. The ME content in SAC should be accurately determined via gas loss assessment in future study. The SAC’s NDF (24.09%) and ADF (8.98%) contents were comparable with those of wheat bran (NDF:32.28%, ADF:11.00%), rice bran (NDF:26.28%, ADF:11.87%), and corn bran (NDF:32.96%, ADF:9.23%). The DE (11.21 MJ/kg) and ME (10.92 MJ/kg) contents in SAC were analogous to wheat bran (DE:10.13 MJ/kg, ME:9.70 MJ/kg), rice bran (DE:12.97 MJ/kg, ME:12.54 MJ/kg), and corn bran (DE:11.08 MJ/kg, ME:10.81 MJ/kg), indicating that SAC could be a fibrous ingredient [19].

In the current study, we observed that pigs fed SAC diets could improve FBW, ADG and reduce F/G. Many studies showed that dietary Se levels and sources did not affect growth performance [14,33,34]. The most likely explanation was that the nutraceutical compounds in SAC, especially the polysaccharide (β-glucan), played a vital role in the growth improvement. The mushrooms’ β-glucan contents ranged from 0.21% to 0.53% (dry weight basis) [35]. As Luo et al. (2019) [36] indicated, β-glucan was a sort of functional polysaccharide that widely existed in the cell wall of fungi and possessed many biological activities. Previous studies showed that pigs fed β-glucan diets might enhance growth performance, which was probably due to the intestinal improvement, as β-glucan could modulate intestinal structure and morphology [30,37,38].

The European Food and Safety Authority (EFSA) suggested that the supplementation level of Se-yeast should not exceed a maximum of 0.2 mg/kg in a complete diet [39]. Therefore, the up-level of Se supplementation in the diet was 0.13 mg/kg in the current study. Most of the researches revealed that growth performance was unaffected by Se levels (0.1, 0.3, and 0.5 mg/kg) and sources (Se-yeast or sodium selenite) [40,41]. A study showed a decreased ADFI of growing-finishing pigs fed 0.5 mg/kg sodium selenite [42]. The discrepancy could be attributed to an adverse effect because inorganic Se is more susceptible to selenosis compared to organic Se [14].

In the present study, pigs fed SAC showed a higher butyrate content compared with the control diet. We speculated that the polysaccharide in SAC might play a central role in increasing SCFA. Polysaccharides could be an effective substrate for the SCFA-generated bacteria and modify the relative composition of microbiota in the intestine. Feed ingredients that stimulate a higher SCFA production, particularly butyrate, were generally considered beneficial [43]. This study was in agreement with previous results, as Högberg et al. (2006) [44] revealed that diets supplemented with high-level insoluble non-starch polysaccharides (NSP) (188–250 g/kg DM) could promote butyrate content in the hindgut. Metzler-Zebeli et al. (2011) [45] also observed that β-glucan increased caecal and colonic butyrate concentrations. The content of NSP and β-glucan in SAC was not detected in this study, and the concentration of crude fiber, NDF, and ADF in SAC was 6.43%, 24.09%, and 8.98%, respectively. A further quantitative study is necessary to explore the mechanism of improving butyrate content by SAC addition.

Butyrate is the result of microbial fermentation in the hindgut of animals. It serves not only as the primary energy source for colonocytes but also as a cellular mediator for gut cells, such as gut tissue development, immune modulation, gene expression, diarrhea control, and oxidative stress reduction [46]. The butyrate transportation was mediated by the monocarboxylate transporter 1 (MCT1). To ascertain the mechanisms, we analyzed the colonic MCT1 mRNA expression, as Cuff et al. (2020) [47] indicated that the MCT1 pathway was positively relevant to the butyrate generation, and butyrate showed a time and concentration-dependent relationship of both MCT1 mRNA and protein. The result showed a higher MCT1 mRNA expression in pigs fed SAC diets, consistent with a previous study indicating that dietary β-glucan supplementation could upregulate MCT1 expression in the cecum [30].

We observed a significant increase in GSH-Px content in serum, LDM, and liver of pigs fed SAC diets. The result might be attributed to the Se present in SAC. Pilarczyk et al. (2012) [48] revealed that Se is an integral part of GSH-Px. It works through the reduction of hydrogen peroxide that protects subcellular and cellular membranes from oxidative damage. This result was in accordance with a previous study where pigs showed a significantly higher serum GSH-Px activity as dietary Se level increased, compared with the basal diet [49]. Pigs fed higher SAC level diets revealed a decreased MDA in the liver. Malondialdehyde, one of the most studied lipid peroxidation products, was an important marker for oxidative stress. The MDA concentration is well correlated with the GSH-Px activity, and GSH-Px protects the tissue from peroxidation to keep MDA content in a low status [50]. Zhan et al. (2007) [50] showed that dietary Se supplementation significantly reduced the MDA content in the liver, which was consistent with our findings.

In the current study, the LDM Se contents were improved remarkably in response to the dietary 2.4% SAC supplementation. The reason might be because muscle Se content had a high correlation to dietary Se content. A previous study demonstrated that diets with a high Se level had a greater loin Se content [51]. This result was consistent with previous findings, which reported a significant improving Se retention in both the muscles and glands of growing pigs fed Se diets [52,53]. Researchers confirmed that organic Se was more greatly retained by tissue (muscle and liver) than inorganic Se [40,49,52]. We have not saturated the mechanism for Se deposition in the growing pigs fed SAC diets, and we observed a lower Se content in muscle than in the other tissue. The possible reason might be the lower rate of muscle accretion, which allowed more Se to be available to be distributed to other non-muscle tissues [40,53].

In this study, SAC-supplemented diets did not affect the meat quality of finishing pigs. The possible reason might be the relatively low Se content (upper limit 0.13 mg/kg) in experimental diets. In the study of Chen et al. (2019) [14], a higher level of dietary Se supplementation (0.5 mg/kg) tended to reduce the drip loss of loins. Similarly, Calvo et al. (2016) [54] reported that a dose of dietary 0.4 mg/kg Se (Se-enriched yeast) addition could increase water-holding capacity. Meat color is one of the leading factors affecting meat purchasing. In the current study, no effect was observed on meat color for pigs fed SAC diets. A relatively low Se level in SAC diets might lead to the result. A supplemented level of 0.5 mg/kg Se could improve a* value of meat in finishing pigs [14]. Calvo et al. (2017) [55] also reported that dietary 0.3 mg/kg Se addition increased the a* value of muscle in growing-finishing pigs. In these studies, the meat color improvement might be attributed to the enhanced antioxidant activities induced by dietary Se supplementation. As Mancini et al. (2005) [56] indicated, Metmyoglobin reduction is a vital factor for meat color life, and remarkably lies on oxygen scavenging enzymes, reducing enzyme systems, and the nicotinamide adenine dinucleotide (NADH) pool.

Lipid peroxidation is a leading cause of meat quality deterioration during storage. In the current study, SAC supplementation reduced MDA content in fresh meat on day 6. Malondialdehyde was generated by lipid oxidation reaction induced by oxygen radicals in tissues and was one of the most crucial metabolites of lipid peroxides [50,57]. The MDA content in meat has been used as an important marker of lipid oxidation intensity [58]. The decreased MDA concentration indicated that Se contained in SAC could enhance the ability to protect against oxidation, delay the onset of rancidity resulting from lipid oxidation, and extend the shelf life of meat to some extent, which could also avoid reducing consumer acceptability. The Se protective action against the deterioration of meat quality during storage was largely attributed to its effects of maintaining muscle membrane integrity and preventing lipid peroxidation [50,57]. Calvo et al. (2017) [59] reported that pigs fed organic Se showed higher stability against oxidation in muscles, which was in accord with our result. Pappas et al. (2012) [57] also found that chickens fed Se diets showed a linear decrease in lipid oxidation of breast muscle, which was probably due to the Se antioxidant properties.

One of the main challenges facing the meat industry is to add value to the final product. The novel Se source might benefit the meat industry because of health improvements and increasing meat oxidative stability. Meat is a major source of nutrition, such as protein, selenium, and vitamins. These nutrients are linked to the maintenance of health status. Research on selenium (particularly organic Se) is increasing rapidly concerning immune response and cancer prevention in humans. It is essential for the meat industry that meat products are delivered to the market not only in terms of consumer preference but also in terms of health benefits. The SAC is mainly composed of fungal polysaccharides, organic Se, and metabolites of AC. Currently, dietary SAC addition increased Se’s content in meat and benefited pigs’ health. In the future, quantification research of SAC is essential for achieving its desired potential benefits in the commercial production and meat industry.

## 5. Conclusions

Dietary SAC supplementation improved growth performance, fecal butyrate content, antioxidant status of serum and tissues, and oxidative stability of fresh meat. These results confirmed the efficacy of SAC, and SAC could be served as a promising alternative feed ingredient. The recommended use of SAC is 2.4% for growing-finishing pigs.

## Figures and Tables

**Table 1:** Analyzed nutrient content of Selenium *Auricularia cornea* culture (%, as-fed basis) ^a^.

Item	SAC ^b^
Dry matter	90.11
Gross energy, MJ/kg	17.38
Crude protein	16.45
Ether extract	4.27
Ash	4.35
Crude fiber	6.43
Neutral detergent fiber	24.09
Acid detergent fiber	8.98
Total phosphorus	1.00
Calcium	0.28
Organic selenium, mg/kg	5.39
Inorganic selenium, mg/kg	0

^a^ All analyzed values were the results in duplicate. ^b^ Selenium *Auricularia cornea* culture.

**Table 2:** Ingredients and analyzed nutrient levels of experimental diets used in Exp. 1 (%, as-fed basis).

Item	Basal Diet	SAC Diet
Ingredient		
Corn	97.40	73.50
SAC ^a^	-, ^c^	24.35
Sodium chloride	0.30	0.30
Limestone	0.90	0.90
Dicalcium phosphate	0.90	0.90
Vitamin and mineral premix ^b^	0.50	0.50
Analyzed nutrient levels		
Dry matter	89.32	90.04
Crude protein	8.33	8.21
Ash	3.53	5.48

^a^ SAC, Selenium *Auricularia cornea* culture; ^b^ Vitamin and mineral premix provided the following per kilogram of diet: vitamin A, 5512 IU; vitamin D_3_, 2200 IU; vitamin E, 30 IU; vitamin B_12_, 27.6 μg; vitamin K_3_, 2.2 mg; riboflavin, 4 mg; D-pantothenic acid, 14 mg; pyridoxine, 3 mg; biotin, 44 μg; folic acid, 0.7 mg; thiamine, 1.5 mg; Fe, 75 mg; Mn 40 mg; Cu, 100 mg; Zn, 75 mg; Se, 0.3 mg and I, 0.35 mg. ^c^, -, None.

**Table 3:** Ingredients and calculated nutrient levels of the treatment diets used in Exp. 2 (%, as-fed basis).

Item	Day 0–23				Day 24–45			
	Control	0.6% SAC	1.2% SAC	2.4% SAC	Control	0.6% SAC	1.2% SAC	2.4% SAC
Ingredients								
Corn	77.61	77.61	77.61	77.52	77.91	77.91	77.87	77.82
Soybean meal	15.00	15.00	15.00	15.00	13.00	13.00	13.00	13.00
Wheat bran	4.00	3.40	2.80	1.60	6.00	5.40	4.80	3.60
Soybean oil	0.70	0.70	0.70	0.74	0.80	0.80	0.82	0.84
SAC ^a^	-	0.60	1.20	2.40	-, ^c^	0.60	1.20	2.40
Dicalcium phosphate	0.60	0.60	0.62	0.66	0.42	0.42	0.42	0.46
Limestone	0.90	0.90	0.88	0.86	0.84	0.84	0.84	0.82
Sodium chloride	0.35	0.35	0.35	0.35	0.35	0.35	0.35	0.35
L-lysine	0.27	0.27	0.27	0.28	0.16	0.16	0.17	0.17
DL-Methionine	-	-	-	0.01	-	-	-	-
L-Threonine	0.06	0.06	0.06	0.07	0.02	0.02	0.02	0.03
L-Tryptophan	0.01	0.01	0.01	0.01	-	-	0.01	0.01
Vitamin mineral premix ^b^	0.50	0.50	0.50	0.50	0.50	0.50	0.50	0.50
Analyzed Nutrient Level								
Dry matter	88.31	89.45	90.12	89.87	89.45	88.78	89.78	89.06
Crude protein	13.40	13.34	13.42	13.50	13.12	13.09	13.19	13.24
Gross energy, MJ/kg	16.24	16.29	16.17	16.22	15.91	16.12	15.81	15.79
Ash	3.94	3.88	4.12	4.07	4.25	4.33	4.30	4.22
Calculated Nutrient Level								
DE, MJ/kg	14.23	14.23	14.23	14.23	14.23	14.23	14.23	14.23
SID Lys	0.73	0.73	0.73	0.73	0.61	0.61	0.61	0.61
SID Met	0.21	0.21	0.21	0.21	0.20	0.20	0.20	0.20
SID Thr	0.46	0.46	0.46	0.46	0.40	0.40	0.40	0.40
SID Trp	0.13	0.13	0.13	0.13	0.12	0.12	0.12	0.12

^a^ SAC, Selenium *Auricularia cornea* culture. ^b^ Premix provided the following per kg of complete diet: vitamin A, 5512 IU; vitamin E, 30 IU; vitamin D_3_, 2200 IU; vitamin B_12_, 27.6 μg; vitamin K_3_, 2.2 mg; thiamine, 1.5 mg; D-pantothenic acid, 14 mg; riboflavin, 4 mg; folic acid, 0.7 mg; pyridoxine, 3 mg; biotin, 44 μg; Fe, 75 mg; Cu, 100 mg; Mn 40 mg; Se, 0.3 mg; I, 0.35 mg and Zn, 75 mg. DE, digestible energy; SID, standardized ileal digestible; Lys, lysine; Thr, threonine; Met, methionine; Trp, tryptophan. ^c^, -, None.

**Table 4:** Primer sequences for real-time polymerase chain reaction analysis.

Item	Primer	Sequence (5′–3′)
β-actin	Forward	TGCGGGACATCAAGGAGAAGC
	Reverse	ACAGCACCGTGTTGGCGTAGAG
MCT1	Forward	GGAGACCAGTATAGACGCTGC
	Reverse	CTCCTCCTCTTTGGGGCTTC

**Table 5:** Energy content (MJ/kg) in Selenium *Auricularia cornea* culture (Exp. 1) ^a^.

Item	SAC
Energy content, MJ/kg, as-fed basis	
Gross energy	17.38
Digestible energy	11.21
Metabolizable energy	10.92
Energy content, MJ/kg, dry matter basis	
Gross energy	19.29
Digestible energy	12.44
Metabolizable energy	12.12

^a^ values are the means of six observations. SAC, Selenium *Auricularia cornea* culture.

**Table 6:** Effects of dietary Selenium *Auricularia cornea* culture supplementation on growth performance of growing-finishing pigs (Exp. 2).

Item	Control	0.6% SAC	1.2% SAC	2.4% SAC	SEM	*p*-Value
IBW	92.02	91.94	91.93	91.96	0.08	0.86
FBW	131.03 ^b^	131.16 ^b^	131.57 ^a,b^	131.97 ^a^	0.21	0.03
Day 1 to 23						
ADG, g	904	911	909	914	3.00	0.18
ADFI, g	2450	2325	2434	2431	34.84	0.08
F/G	2.71 ^a^	2.55 ^b^	2.68 ^a,b^	2.66 ^a,b^	0.03	0.03
Day 24 to 45						
ADG, g	828 ^b^	830 ^b^	852 ^a,b^	863 ^a^	7.19	<0.01
ADFI, g	2525	2515	2508	2527	19.75	0.90
F/G	3.05 ^a^	3.03 ^a^	2.94 ^b^	2.93 ^b^	0.02	<0.01
Day 1 to 45						
ADG, g	867 ^b^	872 ^b^	881 ^a,b^	889 ^a^	3.98	0.01
ADFI, g	2486	2418	2470	2478	18.17	0.07
F/G	2.87 ^a^	2.77 ^b^	2.80 ^b^	2.79 ^b^	0.02	0.02

^a,b^ Least squares means within different superscripts differ (*p* < 0.05). SAC, Selenium *Auricularia cornea* culture; IBW, initial body weight; FBW, final body weight; ADG, average daily gain; ADFI, average daily feed intake; F/G, feed to gain ratio; SEM, standard error of the mean.

**Table 7:** Effects of dietary Selenium *Auricularia cornea* culture supplementation on short chain fatty acid content measured in fresh feces of pigs (mg/kg, Exp. 2).

Item	Control	0.6% SAC	1.2% SAC	2.4% SAC	SEM	*p*-Value
Day 23						
Acetate	4256.34	4244.82	4492.03	4401.33	193.34	0.77
Propionate	3319.74	3391.75	3537.26	3573.86	209.45	0.81
Butyrate	488.09	496.27	496.86	519.22	21.23	0.76
Total	8064.16	8132.85	8526.15	8494.41	282.00	0.55
Day 45						
Acetate	4895.11	4888.38	5172.97	5142.44	95.44	0.09
Propionate	3748.29	3751.36	3765.11	3800.91	118.88	0.99
Butyrate	601.65 ^b^	662.35 ^a,b^	715.22 ^a^	711.80 ^a^	23.04	0.01
Total	9245.05	9302.10	9653.30	9655.16	125.29	0.06

^a,b^ Least squares means within different superscripts differ (*p* < 0.05). SAC, Selenium *Auricularia cornea* culture; SEM, standard error of the mean, *n* = 6.

**Table 8:** Effects of dietary Selenium *Auricularia cornea* culture supplementation on serum profile of pigs in Exp. 2.

Item	Control	0.6% SAC	1.2% SAC	2.4% SAC	SEM	*p*-Value
Day 45						
GSH-Px, U/mL	1109.25 ^a,b^	1050.52 ^b^	1173.73 ^a^	1183.68 ^a^	28.22	0.02
T-AOC, U/mL	13.43	14.26	14.63	15.79	0.84	0.29
SOD, U/mL	75.06	79.49	84.89	79.72	5.05	0.60
MDA, nmol/mL	2.65	2.64	2.83	2.33	0.27	0.61
HDL, mmol/L	1.02	1.08	1.13	1.19	0.12	0.78
LDL, mmol/L	1.31	1.18	1.27	1.13	0.14	0.79
TC, mmol/L	2.49	2.67	2.42	2.57	0.25	0.92
TG, mmol/L	0.56	0.61	0.56	0.64	0.07	0.79
GLU, mmol/L	2.96	2.87	2.83	2.76	0.07	0.30
IL-1β, μg/L	27.21	28.71	27.66	25.13	1.93	0.62
IL-2, ng/L	488.20	506.49	484.68	487.61	15.15	0.73
IL-6, μg/L	90.94	93.05	87.10	93.43	4.08	0.68
TNF-α, ng/L	206.56	211.93	221.56	208.87	9.87	0.72

^a,b^ Least squares means within different superscripts differ (*p* < 0.05). SAC, Selenium *Auricularia cornea* culture; SEM, standard error of the mean, *n* = 6; GSH-Px, glutathione peroxidase; T-AOC, total antioxidant capacity; SOD, superoxide dismutase; MDA, malondialdehyde; HDL, high density lipoprotein; LDL, low density lipoprotein; TC, total cholesterol; TG, triglyceride; GLU, glucose; IL-1β, interleukin-1β; IL-2, interleukin-2; IL-6, interleukin-6; TNF-α, tumor necrosis factor-α.

**Table 9:** Effect of dietary Selenium *Auricularia cornea* culture supplementation on liver and LDM antioxidant status of growing-finishing pigs.

Item	Control	0.6% SAC	1.2% SAC	2.4% SAC	SEM	*p*-Value
Liver						
GSH-Px, U/mg prot	91.26 ^b^	94.58 ^a,b^	104.96 ^a,b^	108.74 ^a^	3.87	0.02
T-AOC, U/mg prot	1.21	1.17	1.16	1.37	0.07	0.17
MDA, nmol/mg prot	1.44 ^a^	1.24 ^a,b^	1.09 ^b^	1.02 ^b^	0.1	0.03
LDM						
GSH-Px, U/mg prot	104.61 ^b^	110.30 ^b^	116.71 ^a,b^	129.17 ^a^	4.96	0.02
T-AOC, U/mg prot	2.97	3.21	3.11	3.35	0.13	0.26
MDA, nmol/mg prot	0.35	0.31	0.34	0.30	0.03	0.68

^a,b^ Least squares means within different superscripts differ (*p* < 0.05); SEM, standard error of the mean. SAC, Selenium *Auricularia cornea* culture; SEM, standard error of the mean, *n* = 6; GSH-Px, glutathione peroxidase; T-AOC, total antioxidant capacity; MDA, malondialdehyde; LDM, *longissimus dorsi* muscle.

**Table 10:** Effects of dietary Selenium *Auricularia cornea* culture supplementation on tissue Selenium concentrations (mg/kg).

Item	Control	0.6% SAC	1.2% SAC	2.4% SAC	SEM	*p*-Value
LDM	0.027 ^b^	0.029 ^b^	0.030 ^b^	0.035 ^a^	0.002	0.02
Liver	0.137	0.145	0.156	0.161	0.007	0.09
Kidney	0.660	0.667	0.678	0.702	0.036	0.85

^a,b^ Least squares means within different superscripts differ (*p* < 0.05); SEM, standard error of the mean, *n* = 6. SAC, Selenium *Auricularia cornea* culture; SEM, standard error of the mean; LDM, *longissimus dorsi* muscle.

**Table 11:** Effects of dietary Selenium *Auricularia cornea* culture supplementation on carcass traits and meat quality of pigs in Exp. 2.

Item	Control	0.6% SAC	1.2% SAC	2.4% SAC	SEM	*p*-Value
Carcass Traits						
Carcass length	106.20	106.96	106.74	106.04	0.85	0.85
Hot carcass weight	98.55	99.83	98.22	98.53	1.61	0.90
Dressing percentage	74.78	74.05	74.91	74.95	0.53	0.60
Loin eye area	42.97	44.04	43.62	45.27	2.75	0.94
Backfat thickness	14.83	14.58	14.52	13.86	0.75	0.82
Marbling	1.75	1.58	1.83	2.17	0.24	0.41
Meat Quality						
Cooling loss	21.50	20.04	20.94	20.22	1.06	0.76
Shear force	33.85	33.09	32.89	33.74	1.38	0.95
Drip loss	2.24	2.19	2.28	1.96	0.27	0.83
L * (45 min)	35.61	36.65	36.92	36.49	0.89	0.76
a * (45 min)	3.83	4.13	4.01	4.47	0.20	0.19
b * (45 min)	3.55	3.47	3.46	4.06	0.24	0.28
L * (24 h)	55.58	56.37	54.98	56.21	0.82	0.63
a * (24 h)	10.27	10.66	10.98	11.04	0.24	0.14
b * (24 h)	7.50	7.97	7.40	7.85	0.29	0.49
pH _45 min_	6.72	6.59	6.63	6.56	0.13	0.85
pH _24 h_	5.92	5.90	5.74	6.03	0.08	0.15

Values are means with pooled SEM, *n* = 6. Selenium *Auricularia cornea* culture; pH_45min_, pH value at 45 min after postmortem; pH_24h_, pH value at 24 h after postmortem; L *, lightness, a *, redness, b *, yellowness, Meat color _45 min_, meat color value at 45 min after postmortem, Meat color _24 h_, meat color value at 24 h after postmortem; SEM, standard error of the mean.

**Table 12:** Effects of dietary Selenium *Auricularia cornea* culture supplementation on oxidative stability (malondialdehyde content, nmol/mg prot) of fresh meat during simulated retail display.

Item	Control	0.6% SAC	1.2% SAC	2.4% SAC	SEM	*p*-Value
Day 0	0.14	0.13	0.13	0.12	0.01	0.39
Day 3	0.47	0.42	0.41	0.39	0.02	0.19
Day 6	0.75 ^a^	0.70 ^a,b^	0.68 ^a,b^	0.60 ^b^	0.03	0.03

^a,b^ Least squares means within a row with different superscripts differ (*p* < 0.05). Values are means with pooled SEM, *n* = 6. Selenium *Auricularia cornea* culture.

## Data Availability

The data presented in this study are available on request from the corresponding author. The data are not publicly available due to commercial reasons.
